# High-speed spinning disks on flexible threads

**DOI:** 10.1038/s41598-017-13137-1

**Published:** 2017-10-13

**Authors:** Zi-Long Zhao, Shiwei Zhou, Shanqing Xu, Xi-Qiao Feng, Yi Min Xie

**Affiliations:** 1 Centre for Innovative Structures and Materials, School of Engineering, RMIT University, Melbourne, 3001 Australia; 2 AML & CNMM, Department of Engineering Mechanics, Tsinghua University, Beijing, 100084 China; 3 XIE Archi-Structure Design (Shanghai) Co., Ltd, Shanghai, 200092 China

## Abstract

A common spinning toy, called “buzzer”, consists of a perforated disk and flexible threads. Despite of its simple construction, a buzzer can effectively transfer translational motions into high-speed rotations. In the present work, we find that the disk can be spun by hand at an extremely high rotational speed, e.g., 200,000 rpm, which is much faster than the previously reported speed of any manually operated device. We explore, both experimentally and theoretically, the detailed mechanics and potential applications of such a thread–disk system. The theoretical prediction, validated by experimental measurements, can help design and optimize the system for, e.g., easier operation and faster rotation. Furthermore, we investigate the synchronized motion of multiple disks spinning on a string. Distinctly different twist waves can be realized by the multi-disk system, which could be exploited in the control of mechanical waves. Finally, we develop two types of manually-powered electric generators based on the thread–disk system. The high-speed rotation of the rotors enables a pulsed high current, which holds great promise for potential applications in, for instance, generating electricity and harvesting energy from ocean waves and other rhythmic translational motions.

## Introduction

**Figure 1:**
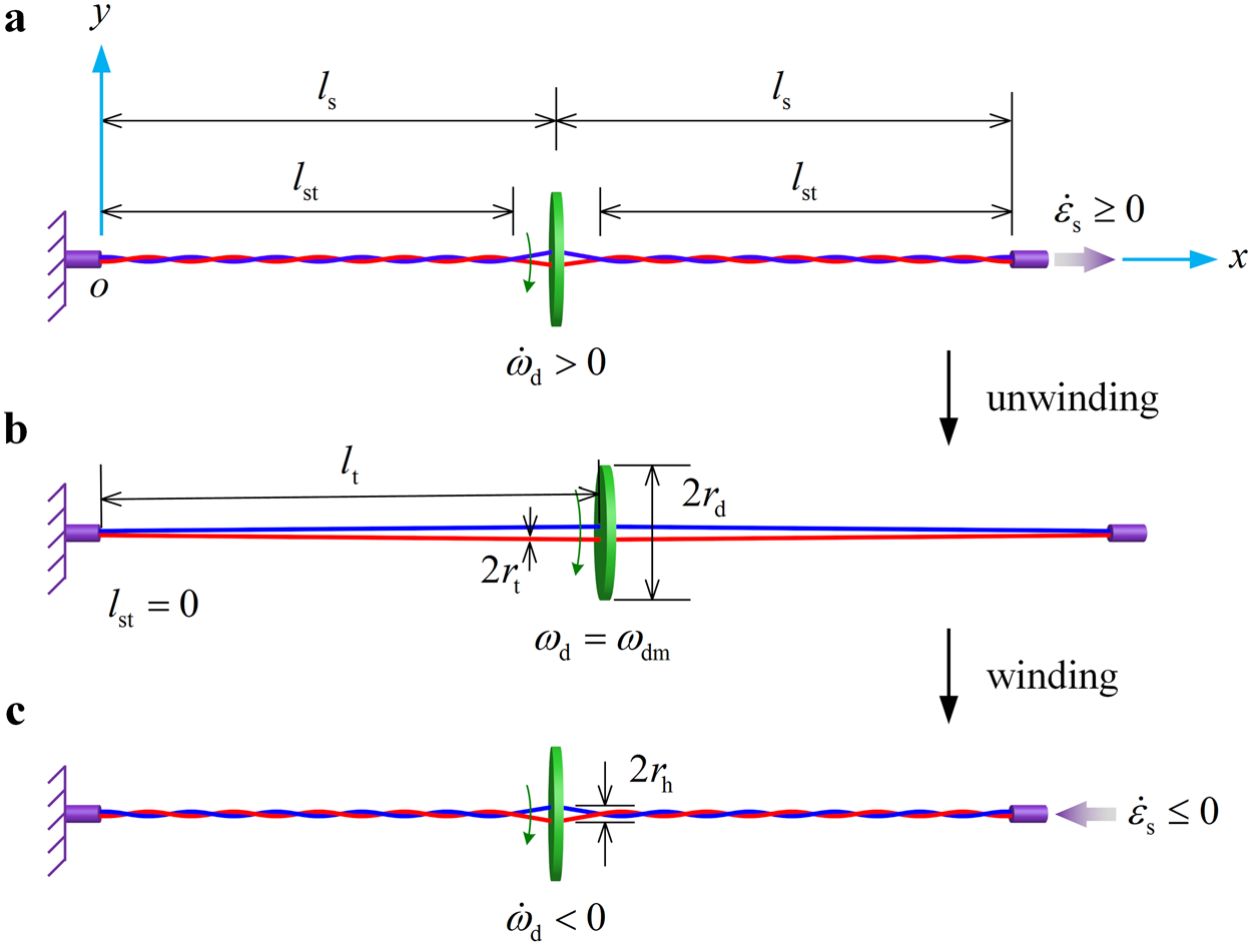
Schematic for theoretical model. Structural parameters of the thread–disk system are illustrated. The periodic rotation of the system is divided into to two stages: unwinding (from (**a**) to (**b**)) and winding (from (**b**) to (**c**)). The Cartesian coordinate system *xoy* is introduced, where the origin *o* is located at the clamped end of the string, and *x* and *y* axes are along and perpendicular to its length direction, respectively. The lengths of the string and its twisted section are denoted as *l*_s_ and *l*_st_, respectively. The length of the threads is denoted as *l*_t_. The radii of the threads and the disk are denoted as *r*_t_ and *r*_d_, respectively. The distance between the center of the disk and the holes is *r*_h_.

Buzzer or whirligig, an ancient mechanical device dating back to more than five thousand years ago^1^, is usually constructed by hanging the perforated rotator at the midpoint of the string, which is made of two flexible threads with the ends tied together (Fig. 1). After winding the string with the ends stationed, the rotation of the disk could be realized by alternately pulling or releasing the tension on it. The kinetic behavior of the system could be described as two successive stages: unwinding and winding. In the unwinding process, the string is subject to axial tensile forces at the two ends, which unwinds the threads and makes the disk spin. The rotational speed of the disk reaches the maximum when the threads are totally unwound. In the winding process, the string is loosened and the momentum of the whirling disk rewinds the threads. When the twist (turns per unit length) of the string increases to the maximum, the motion of the disk comes to a momentary halt. A new rotation cycle of unwinding/winding would start if stretching force is reapplied at this moment.

The thread–disk system is of particular interest owing to its potential applications. The principles of buzzer prototype, featured by its simple construction, can be employed in developing novel devices and materials. For example, one-dimensional or quasi-one-dimensional materials such as carbon nanotube ropes^2,3^ and artificial muscles^4,5^ open opportunities for preparing high-performance thread materials. Rapid development of experimental techniques such as 3D printing and laser engraving enable the easy design and fabrication of the rotators. Resorting to these advancements, many apparatuses such as chemical reactors^6^ and high-speed confocal microscopy systems^7–9^ have been developed recently based on spinning disks. The thread–disk system also shows great potential in producing thin organic films^10–13^, quantitative analysis of cell adhesion^14,15^, high-speed compressive image acquisition^16^, and centrifugation and microfluidics^17–21^.

The dynamic process of the string–disk system involves complicated coupling of different deformation mechanisms, e.g., the tension, bending, and torsion of the threads. The elasticity of the thread materials, a crucial factor of such a system, was not considered in existing studies^22,23^. Experienced operators may find that the unwinding/winding performances of the system significantly depend on, e.g., the positions of the holes on the disk and the rhythm of applied axial forces. Motivated by the promising applications of the system, here we propose a theoretical model to correlate its mechanical responses with the material properties, structural geometries, and loading conditions. The model can be extended to analyze the dynamics of a wide variety of chiral materials^24–26^. Furthermore, we explore the kinetics of spinning multiple disks on a string. The temporal evolutions of the positions and angular velocities of the disks were measured by using a high-speed camera. The influence of the structural parameters and initial conditions on their coupled motion is revealed. Distinctly different twist waves can be realized by using the multi-disk system, which could be used in the control of mechanical waves and the design of, e.g., turbine engines and rotor compressors^27,28^.

Due to the coupled extension and torsion of the helically twisted string^29^, the thread–disk system could effectively transfer the translational motion to a rotational one. At a right rhythm, the disk of a buzzer could be spun at a rotational speed as high as 200,000 rpm, which is the fastest ever experimentally observed speed of any manually operated device (Supplementary Fig. S1), and much higher than the previous record 125,000 rpm^23^. The high-speed rotational motion is of paramount importance in power generation. In 1831, Michael Faraday developed the first homopolar generator, also known as the Faraday disc^30^, which consisted of a conducting flywheel with one electrical contact near the axis and the other near the periphery. When the flywheel was rotated by hand, the generator could produce a current at a low voltage. Inspired by the Faraday disc, we develop two types of thread–disk based electric generators in the present work. Both of them are easily constructed and manually powered. The high-speed rotation of their rotors enables a pulsed high current, which holds great promise for a variety of technologically significant applications in electricity generation.

## Results and Discussion

Figure 1 illustrates the structural parameters of the thread–disk system. Due to symmetry, we consider only the left half of the system. For simplicity, the weight of the disk and threads is neglected in the theoretical model. The force exerted on the string is along the *x* axis. The length of the string is *l*_s0_ in the absence of external loads. The deformed string is *l*_s_ in length, which consists of helically wound threads to the end and unwound threads linking the disk. The length of the twisted string section is denoted as *l*_st_. Let *r*_h_ represent the distance from the center of the disk to the drill holes through which the threads pass. The mass density, Young’s modulus, radius, and winding angle of the threads are denoted as *ρ*_t_, *E*_t_, *r*_t0_, and *φ*_t_, respectively. The mass, radius, thickness, moment of inertia, and angular velocity of the disk are denoted as *m*_d_, *r*_d_, *h*_d_, *J*_d_, and *ω*_d_, respectively.

### Selection of thread materials

The thread–disk device is an energy-dissipative system: in each rotation cycle, the work of the external force is dissipated due to frictions in the threads as well as between the disk and air. Before the system is actuated, the threads are wound up first. The operator may hold the two ends of the string with the disk loosely hanging in the middle and swing in circles to twist the string. Pulling the tightly twisted string, the threads will be unwound and the disk will be twirled. Successive rotation cycles could be achieved only if the momentum of the spinning disk is large enough to rewind the threads; otherwise, the rotation cycle would be aborted as the maximal winding angle *φ*_tm_ of the threads is too small. In order to increase *φ*_tm_, intuitively it is desirable to use a disk with a larger moment of inertia *J*_d_ and threads with lower stiffness in both bending and torsion, i.e., larger-sized disk with greater mass density and more flexible threads. For example, a lightweight small button can hardly be used to wind threads made of relatively stiffer materials such as fishing lines.

Selecting appropriate materials for the threads and disk is a key step in constructing an optimized system. Ideal materials could be easily found for making the disk because only its mass and geometry have significant influence on the mechanical responses of the system. However, for threads, because of the complex coupling of different physical mechanisms in the dynamic process including tension, bending, and torsion, it is rather difficult to select effective and efficient thread materials. During the winding process, the kinetic energy of the disk is converted to the strain energy of the threads. For a certain amount of the total strain energy, threads with lower stiffness will have larger deformation (i.e., a larger winding angle *φ*_tm_). For homogeneous, linearly elastic thread materials, the bending energy *U*_bend_ and torsional energy *U*_torsion_ could be much greater than the tensile energy *U*_tension_. Our theoretical analysis reveals that multi-filament microstructures can effectively reduce the bending and torsional stiffness of the threads, resulting in a larger winding angle *φ*_tm_ (*Supplementary Information*). The larger the filament number, the smaller the normalized energies *U*_bend_/*U*_tension_ and *U*_torsion_/*U*_tension_. When the filament number of the threads is sufficiently large, the internal bending and torsional forces and moments could be neglected. Thus we use the multi-filament cotton/nylon twine as the thread material in this study.

### Unwinding dynamics

During the unwinding process, the string is subjected to coupled extensional–torsional deformations. The axial tensile force *F*_s_ and torque *M*_s_ of the string depend on its axial deformation *ε*_s_, while the torque is transmitted to the disk and twirls it. The kinetic equation of the spinning disk is 2*M*_s_ + *J*_d_d^2^ *φ*_t_/d*t* ^2^ = 0, where the torque *M*_s_ is correlated with the structural geometries and elastic deformation of the threads. With the relation between *M*_s_ and *φ*_t_ determined, the winding angle *φ*_t_ and rotational speed *ω*_d_ of the disk can be solved from the governing equation. The detailed theoretical derivation is given in *Supplementary Information*. Here we attempt to reveal the influence of loading rate ${\dot{\varepsilon }}_{{\rm{s}}}={\rm{d}}{\varepsilon }_{{\rm{s}}}/{\rm{d}}t$ on the unwinding dynamics. In the examples, we take *E*_t_ = 2 GPa, *r*_t0_ = 1 mm, *φ*_tm_ = 3600^o^, *l*_s0_ = 20 cm, *m*_d_ = 50 g, *r*_d_ = 5 cm (*J*_d_ = 6.25 × 10^−5^ kg · m^2^), and *r*_h_ = 1 mm. The strain of the string is assumed to be ${\varepsilon }_{{\rm{s}}}={\varepsilon }_{\min }+({\varepsilon }_{\max }-{\varepsilon }_{\min }){t}^{n}/{t}_{{\rm{unwind}}}^{n}$, where *t*_unwind_ is the unwinding period and *n* a dimensionless exponent. The minimal ${\varepsilon }_{\min }$ and the maximal axial strain ${\varepsilon }_{\max }$ of the string are determined from *F*_s_|_*t*=0 s_ = 0 N and *t*_unwind_ = 1 s.

**Figure 2:**
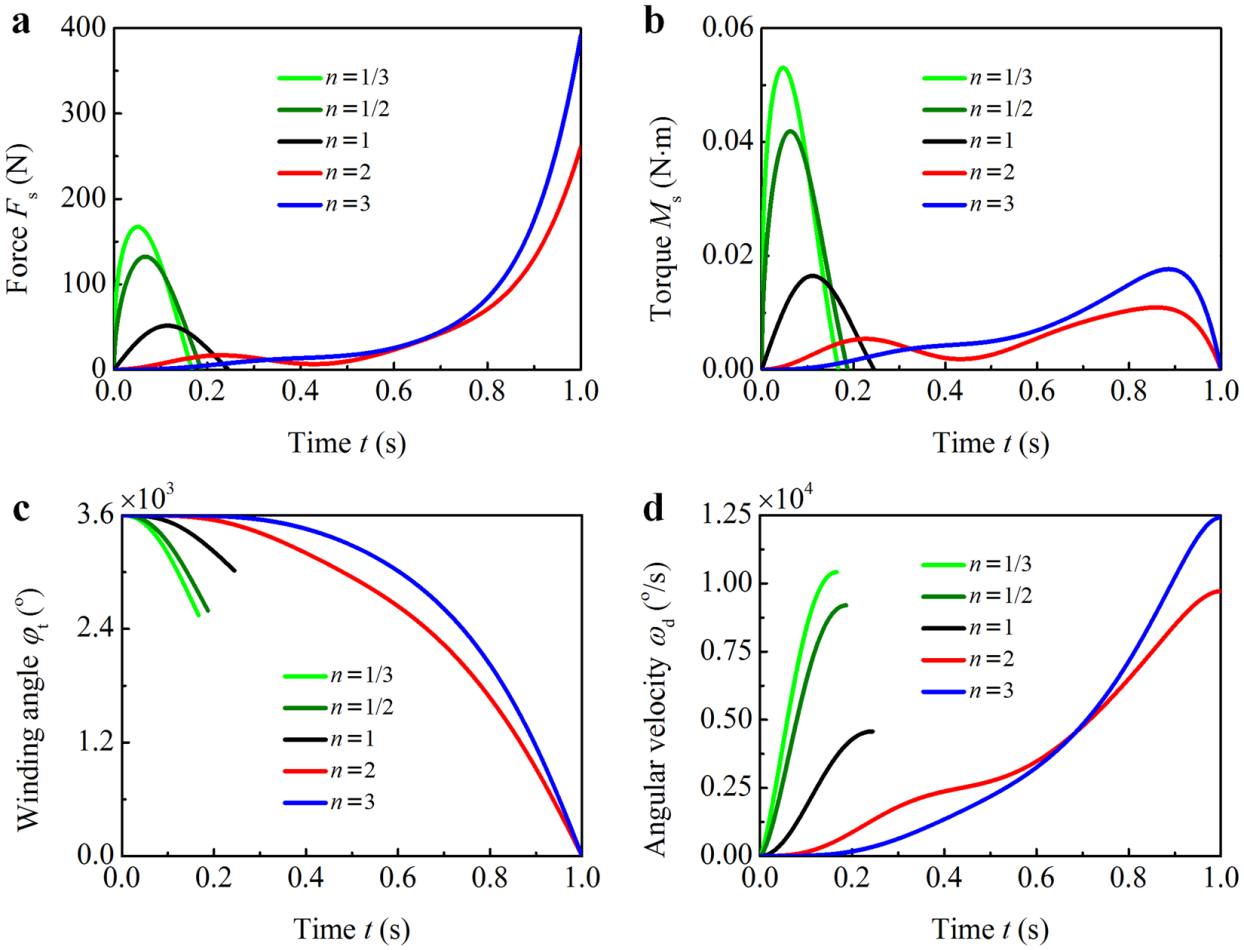
Influence of the loading rate on the unwinding dynamics. (**a**) Tensile force *F*_s_ and (**b**) torque *M*_s_ of the string, (**c**) winding angle *φ*_t_ of the threads, and (**d**) angular velocity *ω*_d_ of the disk are plotted with time *t* for different loading rates ${\dot{\varepsilon }}_{{\rm{s}}}=n({\varepsilon }_{\max }-{\varepsilon }_{\min }){t}^{n-1}/{t}_{{\rm{unwind}}}^{n}$, where *n* is set as 1/3, 1/2, 1, 2, and 3.

The temporal evolutions of the axial tensile force *F*_s_ and torque *M*_s_ of the string, the winding angle *φ*_t_ of the threads, and the angular velocity *ω*_d_ of the disk are plotted in Fig. 2, where we take *n* = 1/3, 1/2, 1, 2, and 3 for different loading rates ${\dot{\varepsilon }}_{{\rm{s}}}$. The maximal axial strain of a string that makes *F*_s_ = 0 N and *M*_s_ = 0 N · m is referred to as the critical strain: ${\varepsilon }_{{\rm{cr}}}=\sqrt{1-{\bar{r}}_{{\rm{t0}}}^{2}{\phi }_{{\rm{t}}}({\phi }_{{\rm{t}}}+2{\bar{r}}_{{\rm{h}}}/{\bar{r}}_{{\rm{t0}}}-2)}-1$. Only when *ε*_s_ > *ε*_cr_, can the string accelerate the disk. As shown in Fig. 2, the unwinding processes of *n* = 1/3, 1/2, and 1 are aborted as a result of *ε*_s_ < *ε*_cr_, while that of *n* = 2 and 3 are completed with ${\phi }_{{\rm{t}}}{|}_{t={t}_{{\rm{unwind}}}}=0^\circ$. In the early stage of the unwinding process, the strain rates ${\dot{\varepsilon }}_{{\rm{s}}}$ in the case of *n* ≤ 1 are distinctly greater than that of *n* > 1, which lead to a rapid decrease in the winding angle *φ*_t_ (Fig. 2c) and, in turn, increase the critical strain *ε*_cr_. If the increasing *ε*_cr_ exceeds *ε*_s_, the string is loosened and featured by *F*_s_ = 0 N and *M*_s_ = 0 N · m (Fig. 2a and b). An appropriate strain rate ${\dot{\varepsilon }}_{{\rm{s}}}$ (e.g., *n* = 2 and 3) enables the disk to be continuously accelerated to the end of the unwinding process (Fig. 2d). It is worthy of mentioning that, less than 200 N is required to twirl the disk to 10000^o^/s (about 1667 rpm) when *n* = 1/3, while nearly 400 N is required in the case of *n* = 3 to make the disk spin at the similar speed (Fig. 2a and d). In the former case, however, the maximal torque *M*_s_ is much larger than that in the later (Fig. 2b).

**Figure 3:**
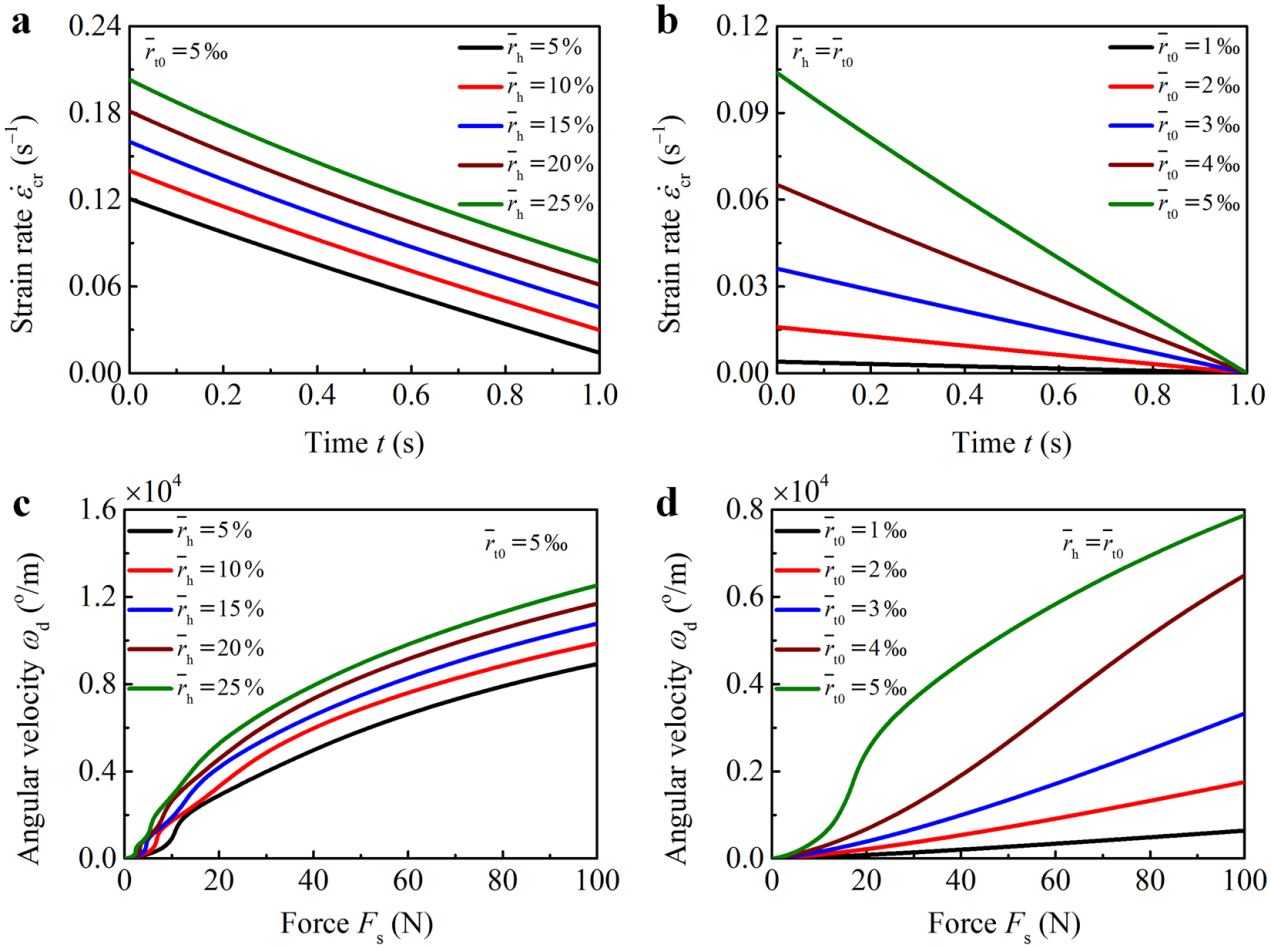
Parametric study of the unwinding dynamics. Critical strain rate ${\dot{\varepsilon }}_{{\rm{cr}}}$ of the string is plotted with time for different ${\bar{r}}_{{\rm{h}}}$ in (**a**) and ${\bar{r}}_{{\rm{t0}}}$ in (**b**). Angular velocity *ω*_d_ of the disk is plotted as a function of the tensile force *F*_s_ of the string for different ${\bar{r}}_{{\rm{h}}}$ in (**c**) and ${\bar{r}}_{{\rm{t0}}}$ in (**d**).

The dependence of the unwinding dynamics on the structural geometries, e.g., the length *l*_s0_ of the string, the radius *r*_t0_ of the threads, and the distance 2*r*_h_ between the drill holes, is also explored. When *ε*_s_ = *ε*_cr_, the disk will be spun at a constant velocity *ω*_c_ and the winding angle of the threads *φ*_t_ = *φ*_tm_ − *ω*_c_ *t*. With *ω*_c_ and *φ*_t_ known, the lower limit of the strain rate that keeps the disk accelerating can be determined as ${\dot{\varepsilon }}_{{\rm{cr}}}={\rm{d}}{\varepsilon }_{{\rm{cr}}}/{\rm{d}}t$. The smaller the ${\dot{\varepsilon }}_{{\rm{cr}}}$, the easier the unwinding process will be. The ${\dot{\varepsilon }}_{{\rm{cr}}}$ vs. *t* relations are plotted in Fig. 3a and b, where we take *φ*_tm_ = 3600^o^, *ω*_c_ = 3600^o^/s, *l*_s0_ = 20 cm, and several representative values of ${\bar{r}}_{{\rm{h}}}={r}_{{\rm{h}}}/{l}_{{\rm{s0}}}$ and ${\bar{r}}_{{\rm{t0}}}={r}_{{\rm{t0}}}/{l}_{{\rm{s0}}}$. The critical strain rate ${\dot{\varepsilon }}_{{\rm{cr}}}$ increases with increasing ${\bar{r}}_{{\rm{h}}}$ and ${\bar{r}}_{{\rm{t0}}}$. It suggests that threads with a large slenderness (i.e., ${\bar{r}}_{{\rm{t0}}}^{-1}={l}_{{\rm{s0}}}/{r}_{{\rm{t0}}}$) and a disk with paracentral holes favor easier unwinding. However, threads with an excessively large length cannot be easily rewound when a lightweight disk is used during the winding process. When the thread radius *r*_t0_ is specified, using longer threads, though with a larger slenderness, may not lead to an easier operation. Excellent performance (e.g., faster rotation and easier operation) of the system requires an adequate combination of thread and disk materials.

The angular velocity *ω*_d_ of the disk is further plotted as a function of the tensile force *F*_s_ of the string in Fig. 3c and d, where we take *E*_t_ = 2 GPa, *φ*_tm_ = 3600^o^, *l*_s0_ = 20 cm, *ε*_s_ = *ε*_cr_|_*t*__=0s_ + *t* ^3^/10, *J*_d_ = 6.25 × 10^−5^ kg · m^2^, and several representative ${\bar{r}}_{{\rm{h}}}$ and ${\bar{r}}_{{\rm{t0}}}$. For a fixed *F*_s_, *ω*_d_ increases with the increasing ${\bar{r}}_{{\rm{h}}}$ and ${\bar{r}}_{{\rm{t0}}}$. This is because the torque transmitted to the disk will be enlarged if ${\bar{r}}_{{\rm{h}}}$ and ${\bar{r}}_{{\rm{t0}}}$ increase. When the length *l*_s0_ of the string is specified, the disk could be spun at a higher speed by using thicker threads, which is consistent with previous experimental observations^1^. It suggests that threads with a smaller slenderness and a disk with a larger distance between drill holes are preferable to achieve a high-speed rotation.

### Winding dynamics

During the winding process, the momentum of the spinning disk rewinds the threads. A mechanics model is proposed to investigate the winding dynamics and the detailed derivation is given in *Supplementary Information*. Denoting the torsional stiffness and the moment of inertia per unit length of the string as *K*_s_ and *J*_s_, respectively, the wave propagation of the twist in the string can be described as ${\partial }^{2}{\omega }_{{\rm{s}}}/\partial {t}^{2}={\lambda }_{{\rm{s}}}^{2}{\partial }^{2}{\omega }_{{\rm{s}}}/\partial {x}^{2}$, where *λ*_s_ = (2π*J*_s_/*K*_s_)^−1/2^ is the propagation velocity of the twist. The initial conditions are assumed to be ${\omega }_{{\rm{s}}}(x,0)={\omega }_{{\rm{dm}}}{x}^{m}/{l}_{{\rm{s}}}^{m}$ and ∂*ω*_s_(*x*,0)/∂*t* = 0, where *ω*_dm_ is the maximal rotational speed of the disk and *m* is a dimensionless exponent. The following parameters are used in the examples: *ρ*_t_ = 1150 kg/m^3^, *E*_t_ = 2 GPa, *r*_t0_ = 1 mm, *l*_s_ = 20 cm, *ε*_s_ = 2%, *ω*_dm_ = 10000^o^/s, and *J*_d_ = 6.25 × 10^−5^ kg · m^2^.

**Figure 4:**
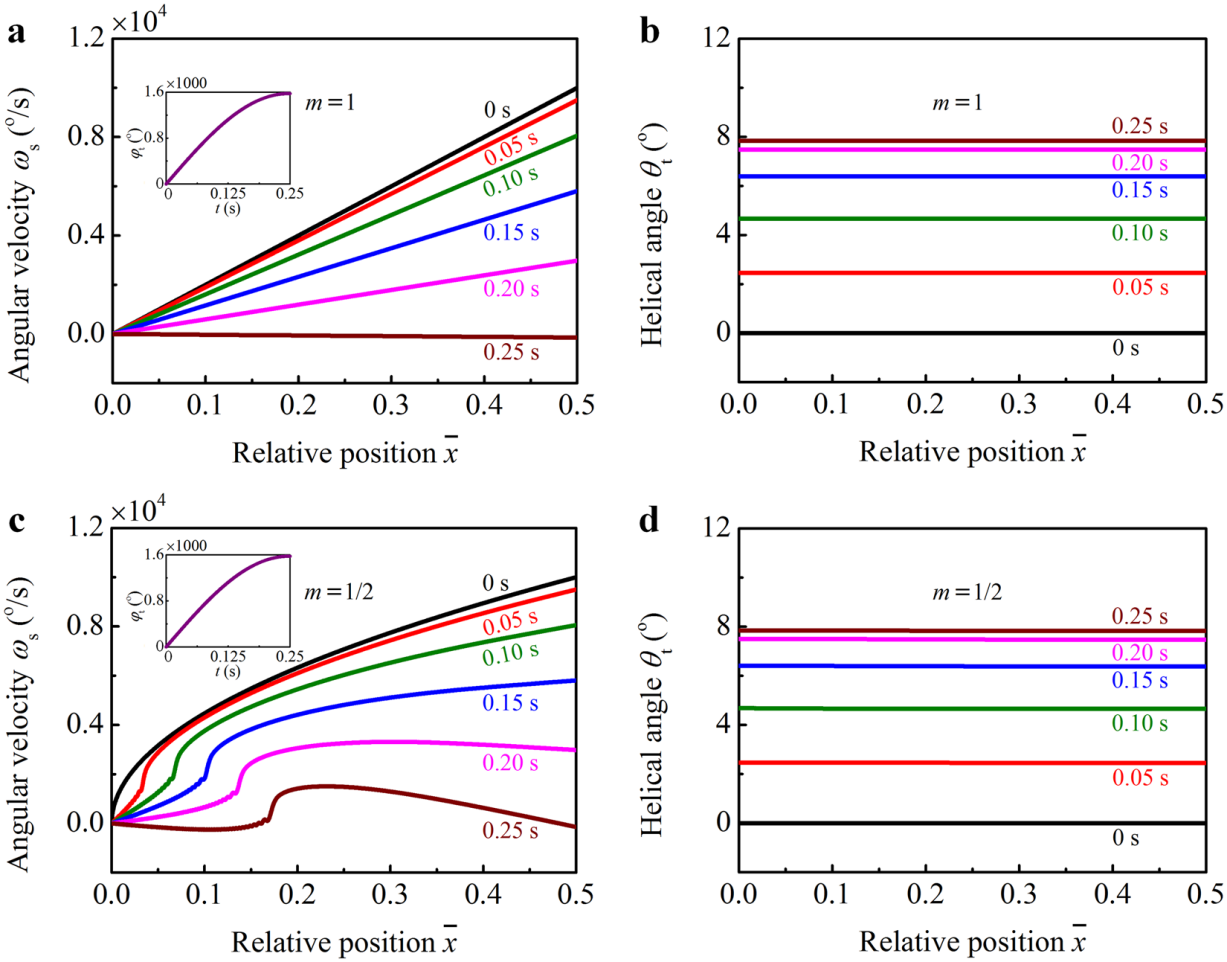
Parametric study of the winding dynamics. Angular velocity *ω*_s_ of the string and helical angle *θ*_t_ of the threads are plotted with $\bar{x}$ for different initial conditions ${\omega }_{{\rm{s}}}(x,0)={\omega }_{{\rm{dm}}}{x}^{m}/{l}_{{\rm{s}}}^{m}$, where *m* = 1 in (**a**) and (**b**), and *m* = 1/2 in (**c**) and (**d**). Temporal evolution of the winding angle *φ*_t_ of the threads is shown in the inserts of (**a**) and (**c**).

The angular velocity *ω*_s_ of the string and the helical angle *θ*_t_ of the threads are plotted with the relative position $\bar{x}=x/(2{l}_{{\rm{s}}})$ in Fig. 4, where we take *m* = 1 in Fig. 4a and b, and *m* = 1/2 in Fig. 4c and d. The *φ*_t_ vs. *t* relations are shown in the inserts of Fig. 4a and c. In the case of *m* = 1, the angular velocity *ω*_s_ increases linearly with $\bar{x}$, while decreases with time *t* (Fig. 4a). The angular acceleration $|{\dot{\omega }}_{{\rm{s}}}|$ of the string increases with *t* as a result of the increasing twist. *ω*_s_ reduces to approximately 0^o^/s when *t* = 0.25 s, suggesting that the kinetic energy of the spinning disk has been totally converted to the elastic strain energy of the threads. Figure 4b shows that the helical angle *θ*_t_ of threads keeps constant along the *x* axis and increases with *t*. Figure 4c shows that *ω*_s_(*x*, *t*) varies nonlinearly with *x* in the case of ${\omega }_{{\rm{s}}}(x,0)={\omega }_{{\rm{dm}}}{x}^{1/2}{l}_{{\rm{s}}}^{-1/2}$. The mechanical constraint at $\bar{x}=0$ interferes with the propagation of the twist wave. After the start of winding, the angular velocity *ω*_s_ of the string has an abrupt decrease near the clamped end. The string keeps rotating when the angular velocity *ω*_d_ = *ω*_s_(*l*_s_, *t*) of the disk decreases to zero (*t* = 0.25 s), suggesting that the kinetic energy of the disk is partly dissipated in the threads. Figure 4d shows a similar *θ*_t_ vs. *x* relation to that in Fig. 4b. It can be seen from Fig. 4 that the initial conditions *ω*_s_(*x*, 0) of the winding process may significantly influence the angular velocity *ω*_s_ of the string.

### Experimental verification

**Figure 5:**
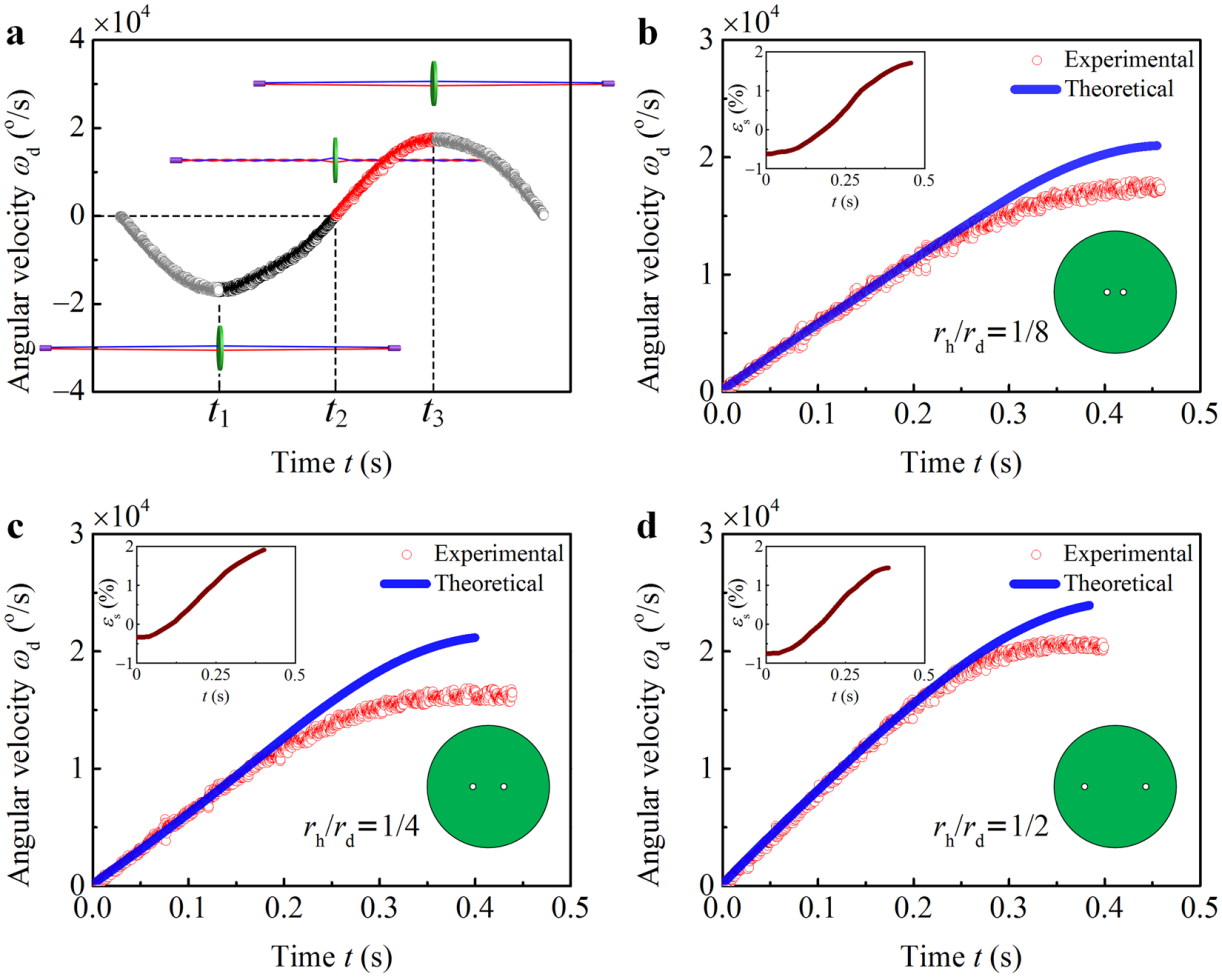
Temporal evolution of the angular velocity of the disk. (**a**) The angular velocity *ω*_d_ of the disk was measured. The threads were wound from *t*_1_ to *t*_2_, and unwound from *t*_2_ to *t*_3_. Both the experimental measurements (red circles) and theoretical predictions (blue lines) of the *ω*_d_ vs. *t* relations are plotted, where *r*_h_/*r*_d_ = 1/8 in (**b**), 1/4 in (**c**), and 1/2 in (**d**). The loading history *ε*_s_(*t*) was measured and given in the insert of each plot.

The angular velocity *ω*_d_ of the disk was measured by using a high-speed camera (Supplementary Video S1). The *ω*_d_ vs. *t* relation is plotted in Fig. 5a, where the inserts illustrate the configurational evolution of the system. The string is totally untwisted (*φ*_t_ = 0^o^) at time *t*_1_ and *t*_3_, and the rotational speed *ω*_d_ peaks at these moments. At *t*_2_, the threads are tightly wound (*φ*_t_ = *φ*_tm_) and the angular velocity *ω*_d_ = 0^o^/s. The maximal winding angle *φ*_tm_ of the threads is calculated by integrating *ω*_d_ from *t*_1_ to *t*_2_. The length *l*_s0_ of the strings used in the experiments was approximately 30 cm. The axial deformation *ε*_s_ of the string was captured by using a digital camera. The parameters of the system were measured as: *E*_t_ = 0.5 GPa, *r*_t0_ = 0.544 mm, *m*_d_ = 8.60 g, *r*_d_ = 2 cm, and *h*_d_ = 6 mm.

The measured *ω*_d_ vs. *t* relations (shown in red circles) are compared with the theoretical predictions (blue lines) in Fig. 5b–d, where the distances 2*r*_h_ between the two drill holes are 5 mm, 10 mm, and 20 mm, respectively. The loading history *ε*_s_(*t*) is shown in the insert of each plot. The theoretical predictions of the angular velocity are in good agreement with the experimental results for small *ω*_d_, while greater than the measurements for large *ω*_d_. This is because the frictional effects in the system and the Poisson effect of the threads are neglected in the theoretical analysis. Due to the friction, the work of external forces cannot be totally converted into the kinetic energy of the disk. The energy dissipated in the threads depends on their complicated deformation, sub-structures, and surface frictional properties. The air drag that tends to decelerate the spinning disk is proportional to ${\omega }_{{\rm{d}}}^{2}$ and will increase rapidly with the increasing radius *r*_d_. Besides, the radius *r*_t_ of the threads shrinks with the increasing tensile strain *ε*_t_. Due to ${M}_{{\rm{s}}}={F}_{{\rm{s}}}{r}_{{\rm{t}}}\,\tan \,{\theta }_{{\rm{t}}}$, neglecting the Poisson effect will lead to an overestimate of the torque that transmitted to the disk. Therefore, the discrepancies between the theoretical predictions and experimental measurements become larger as *ω*_d_ goes higher.

In order to decrease the energy dissipated through the air drag, one may use a disk with a small surface area by reducing the radius *r*_d_ and the thickness *h*_d_. On the other hand, the moment of inertia *J*_d_ of the disk must be large enough to prevent the spinning disk from stalling. It is suggested that materials with higher mass densities are used to maintain the moment of inertia while reducing the size of the disk.

### Multiple disks on a string

**Figure 6:**
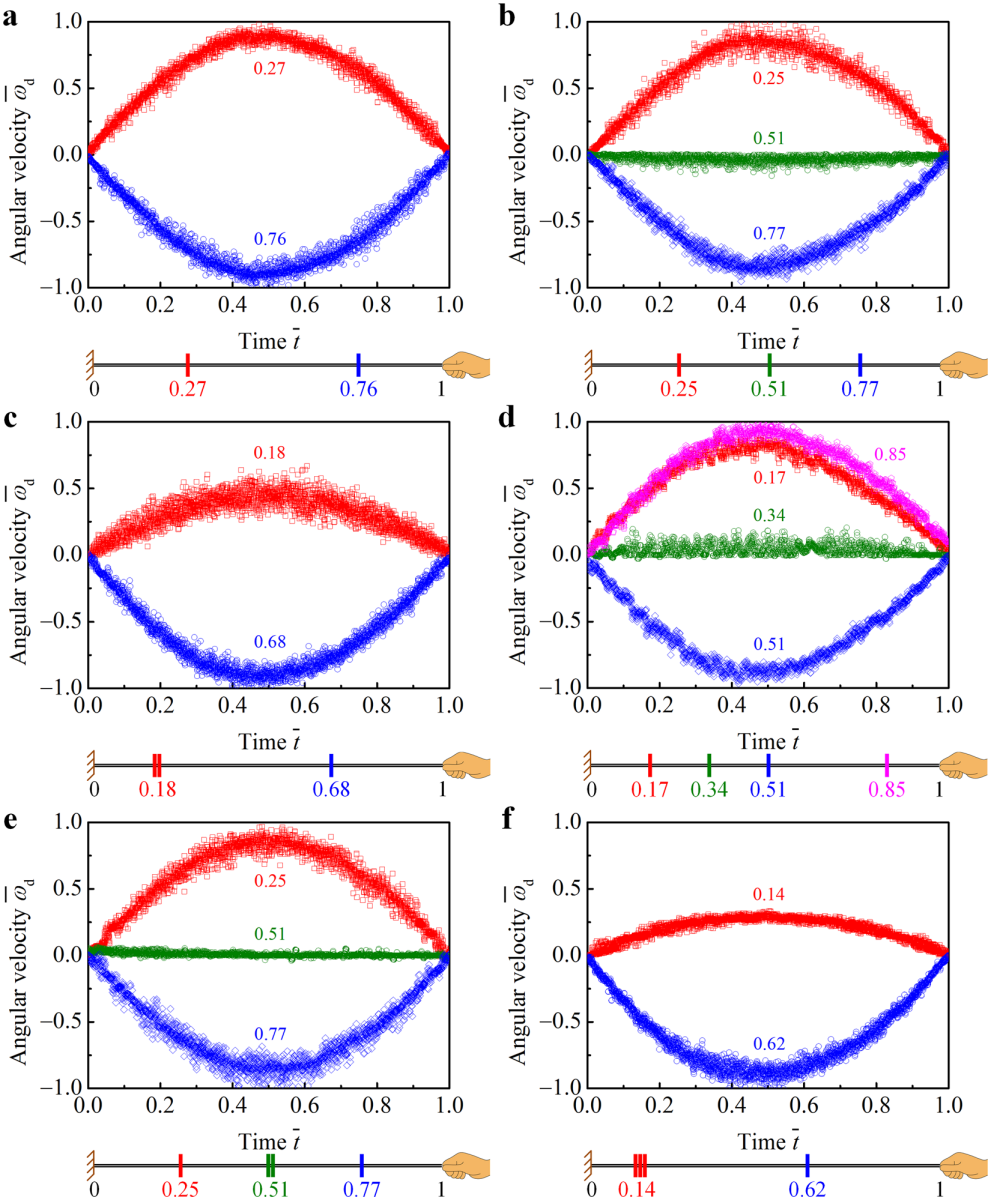
Spinning multiple disks on a string. Temporal evolution of the normalized angular velocity ${\bar{\omega }}_{{\rm{d}}}$ of the disks and their equilibrium positions were measured by using a high-speed camera, with two disks in (**a**), three disks in (**b**) and (**c**), and four disks in (**d**)–(**f**), respectively. All these drawings were drawn by the first author Zi-Long Zhao.

Another set of experiments were performed to investigate the dynamics of spinning multiple disks on a string. The disks were initially located together in the middle of the string. After a few rotation cycles, the disks would move apart from each other. They usually went through multiple instabilities, which would cause the string to wobble. The stability of the system was significantly influenced by the loading conditions (e.g., the frequency of the external force). The dynamic process reaches a steady state only if all disks are trapped in the equilibrium positions; otherwise, they would keep on shifting along the string. Here we analyze the angular velocity and the equilibrium positions of the disks. The parameters of the systems were measured as: *E*_t_ = 0.5 GPa, *r*_t0_ = 0.544 mm, *l*_s0_ = 0.3 m, *m*_d_ = 3.31 g, *r*_d_ = 2 cm, *h*_d_ = 2 mm, and *r*_h_ = 2.5 mm. For each system, let ${\bar{\omega }}_{{\rm{d}}}$ denote the angular velocity normalized by the maximum rotational speed among all disks, and $\bar{t}$ the time normalized by the period of the rotation cycle. Figure 6 shows the ${\bar{\omega }}_{{\rm{d}}}$ vs. $\bar{t}$ relations, where there are two disks in Fig. 6a, three disks in Fig. 6b and c, and four disks in Fig. 6d–f, respectively. The sketch below each plot illustrates the equilibrium positions of the disks. Details of the kinetic behavior of the six systems are demonstrated in Supplementary Videos S2– 9,respectively.

Figure 6a shows that the two disks rotate in opposite directions at the same speed $|{\bar{\omega }}_{{\rm{d}}}|$. The equilibrium positions of the disks are approximately symmetrical about the midpoint of the string ($\bar{x}=1/2$), where the rotational speed of the string is zero. This thread–disk system can be considered as two independent subsystems separated by the midpoint. By neglecting the frictional effects of the thread–disk and thread–thread interfaces, the tensile force of the threads keeps constant along their length directions. The kinetic equilibrium requires that the string have the same amount of twists on both sides of a disk. Thus the ideal equilibrium positions of the two disks should be respectively located at $\bar{x}=1/4$ and 3/4, which are in good agreement with the experimental measurements.

In Fig. 6b, the disk that is trapped in the middle of the string is almost motionless (${\bar{\omega }}_{{\rm{d}}}\equiv 0$). Its influence on the dynamics of the system can be neglected. It is clearly seen that the other two disks exhibit similar dynamic behavior to those in Fig. 6a. In another scenario, as shown in Fig. 6c, two of the three disks, though without glue or constraint between them, stay together and rotate synchronously. Since ${\bar{r}}_{{\rm{h}}}\approx {\bar{r}}_{{\rm{t0}}}$ $\ll$1, the local torque *M*_local_ of the string that is transmitted to the disk is proportional to the local twist *T*_s_, which keeps constant along the length direction of the string (*Supplementary Information*). It is known from d*ω*_d_/d*t* = *M*_local_/*J*_d_ that the angular acceleration of the disk is inversely proportional to its moments of inertia *J*_d_. Thus the rotational speed of the double disks is 1/2 of that of the single disk. Besides, the node (*ω*_s_ ≡ 0^o^/s) of the twist wave is positioned at $\bar{x}=1/3$, such that the string could have a uniform twist. Thus the ideal equilibrium positions of the double disks and the single disk are $\bar{x}=1/6$ and 2/3, respectively, which coincide with the experimental results. It is interesting to note from Fig. 6b and c that a multi-disk system may evolve into different steady states. It suggests that initial conditions, e.g., initial positions of the disks, could significantly influence the dynamics of the system.

Similarly, in Fig. 6d, the disk trapped in the vicinity of the node $\bar{x}=1/3$ and featured by ${\bar{\omega }}_{{\rm{d}}}\equiv 0$ can be neglected in the analysis. The ideal equilibrium positions of the other three disks are $\bar{x}=1/6$, 1/2, and 5/6, respectively. The kinetic behavior of the disks in Fig. 6e and f is similar to those in Fig. 6b and c, respectively. The ideal equilibrium positions of the disks are $\bar{x}=1/4$, 1/2, and 3/4 in Fig. 6e, and $\bar{x}=1/8$ and 5/8 in Fig. 6f. For each system, the whirling disks are coupled by the string: they all end up spinning at the same frequency. Using the above analysis, one can readily predict the possible steady states of the multi-disk system when there are more than 4 disks on a string. The synchronized motion of the disks can be easily tuned by changing, e.g., their number, initial positions, and moments of inertia. Distinctly different twist waves can be realized by the multi-disk system, which may hold potential applications in designing advanced materials and novel devices.

### Electric generators

**Figure 7:**
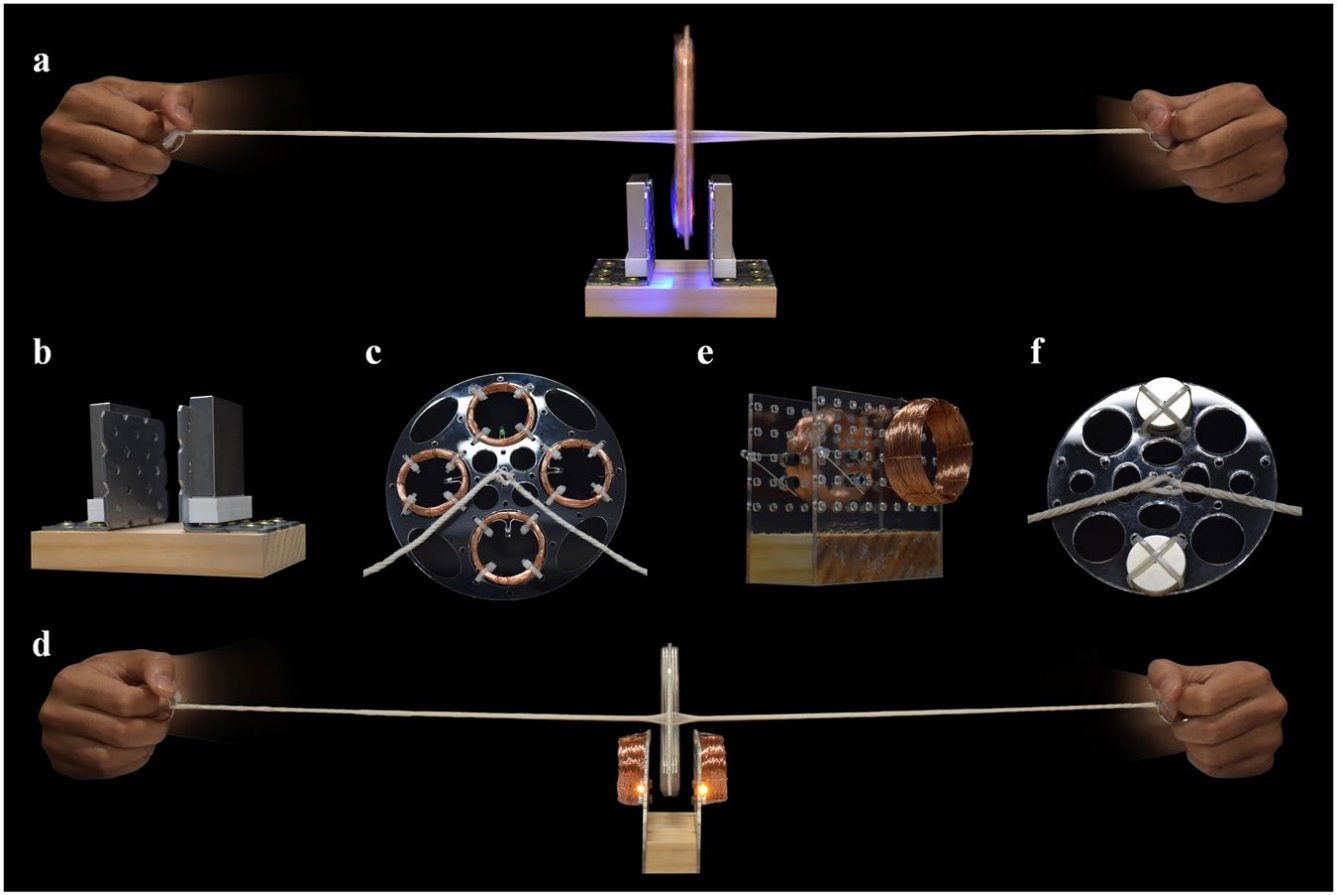
Thread–disk based electric generators. The first generator (**a**) is constructed by the stationary magnets (**b**) and the coils of enameled copper wire (**c**) that are mounted on the spinning disk. The second generator (**d**) is constructed by the stationary coils of wire (**e**) and the lightweight disc magnets (**f**) that are mounted on the perforated disk. All these photographs were taken by the first author Zi-Long Zhao.

The thread–disk system enables a high-speed rotation, which has great potential for power generation use. We developed two types of electric generators based on the system and demonstrated in Fig. 7. Both of them contain permanent magnets and coils of enameled copper wire. They can be easily operated manually to produce alternating current. In the first generator (Fig. 7a), the stator consists of two neodymium block magnets, which are placed parallel to each other (Fig. 7b). The north pole of one magnet faces the south pole of the other. The rotor is comprised of four coils of copper wire mounted on a perforated plastic disk (Fig. 7c). Each of the coils is connected to an LED bulb, forming a closed circuit.

The disk is spun in a plane perpendicular to the uniform static magnetic field between the magnets. The magnetic flux through the wire loop is defined as $\varphi ={\iint }_{{\rm{\Sigma }}}{\boldsymbol{B}}\cdot {\rm{d}}{\boldsymbol{A}}$, where ∑ denotes the hypothetical surface whose boundary is the wire loop, ***B*** the magnetic field, and d***A*** the infinitesimal area element of ∑. Here the magnetic field ***B*** is invariant and the surface ∑ varies with time. The coils rotate with the spinning disk at a high speed in the static magnetic field, rending a rapid change in the magnetic flux *ϕ*. According to Faraday’s law, the voltage between the two ends of a coil is calculated as *U* =  − *N*d*ϕ*/d*t*, where *N* designates the number of turns of the coil. A higher voltage could be generated if the number of turns *N*, the magnetic field ***B***, and the rotational speed *ω*_d_ of the disk increase. When the voltage *U* is sufficiently large, the electric current flowing through the circuit can light the LED bulbs (Fig. 7a and Supplementary Video S8).

In the second electric generator (Fig. 7d), the stator is the coils of wire (Fig. 7e) and the rotor is made of several neodymium disc magnets (Fig. 7f). The coils are immobilized on two vertical plates and the magnets are mounted on a spinning disk. The hypothetical surfaces ∑ of the wire loops are perpendicular to the string. During the rotation cycle, the surface ∑ remains unchanged and the magnetic field ***B*** varies with the whirling magnets. The bulbs connected to the coils can be lit when the rotational speed of the disk is sufficiently high (Fig. 7d and Supplementary Video S9). For example, in this electric generator, the disk is 100 mm in diameter, each coil has 360 turns, and the magnetic flux of each neodymium disc magnet is about 0.1 T. The maximal voltage of the generator can be about $14\,{\rm{V}}$ when the disk rotates at 1000 rpm. The voltage of the bulbs to obtain typical optical characteristics is 1.8–3.3 V. Thus the bulbs can be easily lit by the generator.

The thread–disk based electric generators presented here are easy-to-use and constructed by only a few simple components. They take advantage of the high-speed rotation of the disk. Our experiments demonstrated that the maximal rotational speed of the disk could be as high as 200,000 rpm. Besides, the thread–disk system can effectively transfer the translational force to a rotational motion, which could be exploited to harvest the kinetic energy of wind and ocean waves. The endless movements of wind and water always create enormous amounts of energy^31^. Hydroelectric power stations use the torrent of dammed water to spin the propeller-like turbines by striking their angled blades. Then the generators connected to the turbines convert the rotational motion into electricity. The energy of moving wind is captured and harvested in a similar way for power generation.

When the thread–disk based electric generators are used to capture the wind power, they can be microminiaturized and used in an array. The wind force^32^, *F*_w_ = *C*_d_ *P*_w_ *A*_s_, could be transmitted by tailor-made windshields to drive the generators, where *C*_d_, *P*_w_, and *A*_s_ denote the drag coefficient, wind pressure, and projected area of the windshields, respectively. *C*_d_ is dimensionless and has the order of magnitude 1. When *P*_w_ = 300 Pa (the wind speed is about 22 m/s) and *A*_s_ = 1 m^2^, The wind can make a rotor with *J*_d_ = 5 × 10^−5^ kg · m^2^ spin at approximately 2000 rpm. By optimizing the structural geometries and topologies of the system (e.g., reducing the surface area of the rotors), the energy loss induced by, e.g., air drag, could be reduced and the efficiency of energy conversion could be further improved. The ultrahigh rotational speed of the rotors enables pulsed high currents, which can be applied in, e.g., generating electricity and harvesting energy from ocean waves and other rhythmic translational motions.

## Conclusions

In summary, we have investigated the dynamic behavior of the thread–disk system. A theoretical model is established to examine the dependence of its unwinding and winding performance on the material properties, structural geometries, and loading conditions. The theoretical analysis, validated by experiments, can help design and optimize the system for, e.g., easier operation and faster rotation. The synchronization phenomenon of spinning multiple disks on a string is observed and measured. The influence of the structural parameters, e.g., the number and initial positions of the disks, on their synchronized motion is revealed. Distinctly different twist waves can be realized by the multi-disk system, which is potentially important in designing advanced materials and novel devices, e.g., turbine engines and rotor compressors. Finally, two types of electric generators are developed based on the thread–disk system, which take advantage of the high-speed rotation of the spinning disk. This work has shed light on the mechanics of the thread–disk system and demonstrated an important application in generating electricity. The established theoretical model has paved a way towards further applications of the thread–disk system in various fields. It is also worth mentioning that precisely evaluating the energy dissipation of the system could help improve its efficiency for the energy conversion, which deserves further research.

## Methods

### Measurements of the dynamics of the thread–disk system

Cotton twine and plastic laminates (Polymethylmethacrylate, PMMA) were used for the threads and disks, respectively. The perforated disks were fabricated by using a laser engraving machine (Rayjet-156, Trotec Laser, Austria). Stainless steel key rings were used as handles. Each end of the string was tied to a handle, where one handle was fixed on a support stand and the other was held by hand. The slow motion analysis of the spinning disk was performed by using a high-speed video camera (Fastcam Mini UX100, Photron, Japan). A digital camera (EOS 6D, Canon, Japan) was used to capture the deformation of the string, where a 1000 mm steel ruler was mounted parallel to the string to measure its length. The angular velocity of the disks and the axial tensile strain of the strings were determined through image processing.

### Measurements of mechanical properties of threads

Quasi-static uniaxial tensile tests of the threads were performed with a crosshead speed of 2.0 mm/min at the room temperature using universal testing machine (ESA-CU200, Shimadzu, Japan). The initial distance between clamps in the tensile tests was 0.1 m. The force–displacement curves were recorded automatically. The thread approximately had a circular cross section, and its diameter was measured by using a vernier caliper. The Young’s modulus of the material was determined from the linear regime of the stress–strain curves.

### Spinning the disk at 200,000 rpm

Nylon twine was here used for making the threads to provide high tensile strength and high wear-resistance. The threads were thin and flexible, which could be easily wound by a disk with a small moment of inertia. The radius and the thickness of the disk could be reduced to decrease the air drag. Copper sheet, featured by a high mass density, was used for the disk to maintain the moment of inertia while reducing the surface area. The edges of the holes on the disk were polished by fine sandpaper, which effectively reduced the abrasion to the threads. Pine wood was used for the handles. The parameters of the thread–disk system were measured as: *E*_t_ = 30 GPa, *r*_t0_ = 0.06 mm, *l*_s0_ = 1.5 m, *m*_d_ = 1.39 g, *r*_d_ = 1 cm, *h*_d_ = 0.56 mm, and *r*_h_ = 1.5 mm. The angular velocity of the spinning disk was measured by using a high-speed camera (Fastcam Mini UX100, Photron, Japan), where the video-recording frame-rate and shutter speed were set as 10,000 frames per second and 1/20,000 s, respectively. The maximum recorded rotational speed of the disk was as high as 200,000 rpm (Supplementary Figure S1).

### Electric generators based on the thread–disk system

Coils of enameled copper wire and neodymium magnets were used for both generators. The diameter and linear mass density of the wire are 0.25 mm and 0.44 g/m, respectively. Each of the circular coils, 4 cm in diameter, has approximately 180 turns in the first generator (Fig. 7a) and 360 turns in the second (Fig. 7d). The mass, dimensions, and magnetic flux of each block magnet, used for the first generator, are 231 g, 50 × 50 × 12.5 mm (length × width × thickness), and 0.2598 T, respectively. The mass, diameter, thickness, and magnetic flux of each neodymium disc magnet used for the second generator are 7 g, 25 mm, 2 mm, and 0.0995 T, respectively. The total mass and diameter of the rotors are 68.48 g and 150 mm for the first generator and 78.29 g and 100 mm for the second. LED bulbs with different colors, including red, blue, green, and yellow, were used in the experiments. The continuous forward current and voltage of the bulbs to obtain typical optical characteristics are 20 mA and 1.8–3.3 V, respectively. White nylon cable ties (100 mm in length and 2.5 mm in width) were used to fix the coils of wire and the disc magnets on the disks. Four threads were used to spin the disk in each of the electric generator.

### Data availability

The data that support the findings of this study are available from the corresponding author upon request.

## Electronic supplementary material

SUPPLEMENTARY INFO

Video 1

Video 2

Video 3

Video 4

Video 5

Video 6

Video 7

Video 8

Video 9
