# Supplementary material for: High-speed spinning disks on flexible threads
Source: Sci Rep. 2017 Oct 13;7:13111. doi: 10.1038/s41598-017-13137-1 (PMC5640620; doi:10.1038/s41598-017-13137-1)
Supplement: Supplementary file 1 — SUPPLEMENTARY INFO [file 41598_2017_13137_MOESM1_ESM.pdf]

Supplementary Information for "High-speed spinning disks on flexible threads"

Zi-Long Zhao, Shiwei Zhou, Shanqing Xu, Xi-Qiao Feng, and Yi Min Xie

Detailed theoretical derivations

The detailed derivations pertaining to results and discussion in the main text on the dynamics of the thread–disk system are given below. As shown in Fig. 1, the rotation cycle of the system consists of two successive phases, i.e., unwinding and winding, which are investigated in the first and second subsections, respectively. In the third subsection, how to select flexible materials for the threads is revealed by analyzing their structure–property relation.

The weight and frictional effects of the thread–disk system are neglected. The force exerted on the string is along its length direction. Refer to the Cartesian coordinate system xoy, where the origin o is located at the tied end of the string, and x and y axes are along and perpendicular to the length direction, respectively. Due to symmetry, we consider only the left half of the system. The length of the string is l_{s0} in the absence of external force. Here and in the sequel, the subscript 0 refers to the parameters in the undeformed configuration. The length of the deformed string is l_s which is divided into two parts. In the first part, the threads are helically wound, while in the second part, they assume a straight configuration.

Unwinding dynamics

We first investigate the unwinding process of the system. When subjected to an axial tensile deformation, the twisted part of the string deforms uniformly along the x axis. Denote the axial tensile strain of the string and the winding angle of the threads as ε_s and φ_t, respectively. The axial tensile strain ε_t and the helical angle

θ_t of the threads are expressed in terms of ε_s and φ_t as[1, 2]

$$\varepsilon_t = (1+\varepsilon_s)\frac{\cos\theta_{t0}}{\cos\theta_t} - 1, \tag{1}$$

$$\theta_t = \arctan\frac{\varphi_t r_s}{l_{st}}, \tag{2}$$

where r_s is the helical radius of the string, and l_{st} the length of the twisted part. Let $2r_h$ represent the distance between the two drill holes of the disk. The length l_{st} is written as

$$l_{st} = l_s - (r_h - r_s)\cot\theta_t, \tag{3}$$

where $l_s = l_{s0}(1+\varepsilon_s)$. Substituting Eq. (3) into (2) leads to

$$\theta_t = \arctan\frac{\varphi_t r_s + r_h - r_s}{l_{s0}(1+\varepsilon_s)}. \tag{4}$$

In the undeformed configuration, $\varphi_t = \varepsilon_s = 0$ and $\theta_{t0} = \arctan(r_h/l_0 - r_{s0}/l_0)$. Using the deformation relations in Eqs. (1) and (4), we can further analyze the internal forces of the threads. Consider that the threads are made of flexible materials. As shall be explained later, the internal forces induced by the bending and torsional deformations can be neglected. The tensile force of a thread induced by its axial strain ε_t is

$$F_t = E_t \pi r_t^2 \varepsilon_t, \tag{5}$$

where E_t and r_t denote the Young's modulus and radius of the thread, respectively. The axial force and torque of the string induced by the tensile force F_t are given as

$$F_s = 2F_t\cos\theta_t, \tag{6}$$

$$M_s = 2F_t r_s \sin\theta_t. \tag{7}$$

The helical radius of the string shrinks with the increasing tensile strain: $r_s = r_{s0}(1-\nu_t\varepsilon_t)$, where ν_t is the Poisson's ratio of the thread material. By neglecting the Poisson effect (i.e., $\nu_t = 0$), one has $r_s = r_{s0} = r_{t0}$. Substituting Eqs. (1), (4), and (5) into (6) and (7) yields

$$F_{\rm s} = 2E_{\rm t}\pi r_{\rm t0}^2 \left(1+\varepsilon_{\rm s}\right)\left\{\left[1+\left(\bar{r}_{\rm h}-\bar{r}_{\rm t0}\right)^2\right]^{-1/2} - \left[\left(1+\varepsilon_{\rm s}\right)^2 + \left(\varphi_{\rm t}\bar{r}_{\rm t0}+\bar{r}_{\rm h}-\bar{r}_{\rm t0}\right)^2\right]^{-1/2}\right\}, \quad (8)$$

$$M_{\rm s} = \frac{\varphi_{\rm t}\bar{r}_{\rm t0}+\bar{r}_{\rm h}-\bar{r}_{\rm t0}}{1+\varepsilon_{\rm s}} r_{\rm t0} F_{\rm s}, \quad (9)$$

where the normalized parameters $\bar{r}_{\rm h} = r_{\rm h}/l_{\rm s0}$ and $\bar{r}_{\rm t0} = r_{\rm t0}/l_{\rm s0}$.

The flexible string cannot resist an axial compressive force, i.e., $F_{\rm s} \geq 0$. Therefore, the axial strain $\varepsilon_{\rm s}$ must not be smaller than a critical value

$$\varepsilon_{\rm cr} = \sqrt{1-\bar{r}_{\rm t0}^2\varphi_{\rm t}\left(\varphi_{\rm t}+2\bar{r}_{\rm h}/\bar{r}_{\rm t0}-2\right)} - 1. \quad (10)$$

Provided that the helically twisted threads are not supercoiled, which requires that $\theta_{\rm t} \leq 45^{\rm o}$ [3]. The winding angle $\varphi_{\rm t}$ of the threads cannot exceed a critical value

$$\varphi_{\rm cr} = \bar{r}_{\rm t0}^{-1}\left(1+\varepsilon_{\rm s}-\bar{r}_{\rm h}\right)+1. \quad (11)$$

When the conditions $\varepsilon_{\rm s} \geq \varepsilon_{\rm cr}$ and $\varphi_{\rm t} \leq \varphi_{\rm cr}$ are satisfied, one can derive the kinetic equation of the spinning disk:

$$2M_{\rm s} + J_{\rm d}\frac{{\rm d}^2\varphi_{\rm t}}{{\rm d}t^2} = 0, \quad (12)$$

where $J_{\rm d}$ represents the moment of inertia of the disk. For a circular disk, $J_{\rm d} = m_{\rm d} r_{\rm d}^2/2$, where $m_{\rm d}$ and $r_{\rm d}$ denote its mass and radius, respectively. The initial conditions are given as

$$\varphi_{\rm t}\big|_{t=0} = \varphi_{\rm tm}, \quad (13)$$

$$\frac{{\rm d}\varphi_{\rm t}}{{\rm d}t}\Big|_{t=0} = 0. \quad (14)$$

Once the axial strain $\varepsilon_{\rm s}(t)$ is prescribed, the winding angle $\varphi_{\rm t}$ can be readily determined from Eqs. (12)–(14). For example, we take strain $\varepsilon_{\rm s}$ as

$$\varepsilon_{\rm s} = \varepsilon_{\rm min} + \left(\varepsilon_{\rm max}-\varepsilon_{\rm min}\right)\left(\frac{t}{t_{\rm unwind}}\right)^n, \quad (15)$$

where $t_{\rm unwind}$ is the unwinding period and n a dimensionless exponent. The minimal $\varepsilon_{\rm min}$ and maximal axial strain $\varepsilon_{\rm max}$ of the string are determined from

$F_s|_{t=0\,\mathrm{s}} = 0\ \mathrm{N}$ and $t_{\mathrm{unwind}} = 1\,\mathrm{s}$. At the end of the untwisting process, the rotational speed of the disk reaches the maximum ω_{dm}.

The unwinding dynamics of the thread–disk system depends greatly on the loading rate $\dot{\varepsilon}_s = \mathrm{d}\varepsilon_s / \mathrm{d}t$. The lowest strain rate $\dot{\varepsilon}_{\mathrm{cr}}$ that keeps the disk accelerating is derived. When $\varepsilon_s = \varepsilon_{\mathrm{cr}}$, the disk will rotate with a constant angular velocity ω_c. The process of accelerating the disk from 0 to ω_c is not considered. The winding angle is solved from Eqs. (12) and (13):

$$\varphi_t = \varphi_{\mathrm{tm}} - \omega_c t. \tag{16}$$

Substituting Eq. (16) into (10) results in

$$\varepsilon_{\mathrm{cr}} = \sqrt{1 - \bar{r}_{t0}^2 \left(\varphi_{\mathrm{tm}} - \omega_c t\right)\left(\varphi_{\mathrm{tm}} - \omega_c t + 2\bar{r}_h / \bar{r}_{t0} - 2\right)} - 1. \tag{17}$$

The lower limit of the strain rate is then determined as $\dot{\varepsilon}_{\mathrm{cr}} = \mathrm{d}\varepsilon_{\mathrm{cr}} / \mathrm{d}t$.

Winding dynamics

When the string is totally untwisted, the momentum of the disk will rewind the threads. Assuming that $\bar{r}_h \approx \bar{r}_{t0}$, the kinematic equation of twisting writes[4]

$$\frac{\partial \omega_s}{\partial x} = 2\pi \frac{\partial T_s}{\partial t}, \tag{18}$$

where ω_s and T_s denote the angular velocity and local twist (i.e., turns per unit length) of the string, respectively. The kinetic equation of string twisting is given as[5]

$$J_s \frac{\partial \omega_s}{\partial t} = \frac{\partial M_{\mathrm{local}}}{\partial x}, \tag{19}$$

where J_s and M_{local} are the moment of inertia per unit length and the local torque of the string, respectively. Let ρ_t represent the mass density of the threads. One has

$$J_s = 2\rho_t \pi r_t^2 \left(\frac{1}{2} r_t^2 + r_s^2\right) = 3\rho_t \pi r_{t0}^4. \tag{20}$$

In the following, we consider two cases. The first is a special case where the strain ε_s of the string is a constant ε_c ($\varepsilon_c > \varepsilon_{\mathrm{cr}}$), while in the second case,

$M_{\mathrm{local}} = 0$.

Assuming that $\bar{r}_{\mathrm{h}} \approx \bar{r}_{\mathrm{t0}} \ll 1$, Eq. (9) is rewritten as

$$M_{\mathrm{s}} = \frac{\varphi_{\mathrm{t}} \bar{r}_{\mathrm{t0}}}{1+\varepsilon_{\mathrm{s}}} r_{\mathrm{t0}} F_{\mathrm{s}} = 2E_{\mathrm{t}} \pi r_{\mathrm{t0}}^3 \bar{r}_{\mathrm{t0}} \varphi_{\mathrm{t}} \left\{ 1 - \left[\bar{r}_{\mathrm{t0}}^2 \varphi_{\mathrm{t}}^2 + \left(1+\varepsilon_{\mathrm{s}}\right)^2 \right]^{-1/2} \right\}. \tag{21}$$

The winding angle φ_{t} of the threads is calculated by

$$\varphi_{\mathrm{t}} = 2\pi \int_0^{l_{\mathrm{s}}} T_{\mathrm{s}} \mathrm{d}x . \tag{22}$$

When $\varepsilon_{\mathrm{s}} = \varepsilon_{\mathrm{c}}$ and $\bar{r}_{\mathrm{t0}}^2 \varphi_{\mathrm{t}}^2 \ll 1$, one can derive the local torque of the string as

$$M_{\mathrm{local}} = 4E_{\mathrm{t}} \pi^2 r_{\mathrm{t0}}^4 \varepsilon_{\mathrm{c}} T_{\mathrm{s}} . \tag{23}$$

This result is in agreement with solutions in previous studies[6, 7]. The M_{local} vs T_{s} relation is linearized as $M_{\mathrm{local}} = K_{\mathrm{s}} T_{\mathrm{s}}$, where $K_{\mathrm{s}} = 4E_{\mathrm{t}} \pi^2 r_{\mathrm{t0}}^4 \varepsilon_{\mathrm{c}}$ is the torsional stiffness. The kinetic equation (19) is then simplified as

$$J_{\mathrm{s}} \frac{\partial \omega_{\mathrm{s}}}{\partial t} = K_{\mathrm{s}} \frac{\partial T_{\mathrm{s}}}{\partial x} . \tag{24}$$

From Eqs. (18) and (24), the equation of wave propagation can be derived as

$$\frac{\partial^2 \omega_{\mathrm{s}}}{\partial t^2} = \lambda_{\mathrm{s}}^2 \frac{\partial^2 \omega_{\mathrm{s}}}{\partial x^2} , \tag{25}$$

where the parameter

$$\lambda_{\mathrm{s}} = \sqrt{\frac{K_{\mathrm{s}}}{2\pi J_{\mathrm{s}}}} \tag{26}$$

denotes the propagation velocity of the twist. The initial conditions are assumed to be

$$\omega_{\mathrm{s}}\left(x,0\right) = \left(\frac{x}{l_{\mathrm{s}}}\right)^m \omega_{\mathrm{dm}} , \tag{27}$$

$$\frac{\partial \omega_{\mathrm{s}}\left(x,0\right)}{\partial t} = 0 , \tag{28}$$

where m is a dimensionless exponent. The torque of the string that is transmitted to the disk is calculated by

$$M_{\rm s}=K_{\rm s}\hat{T}_{\rm s}=\frac{K_{\rm s}}{2\pi l_{\rm s}}\int_0^t \omega_{\rm s}\left(l_{\rm s},\tau\right)\mathrm{d}\tau\,, \tag{29}$$

where $\hat{T}_{\rm s}$ represents the average twist of the string. The boundary conditions can then be derived as

$$\omega_{\rm s}\left(0,t\right)=0\,, \tag{30}$$

$$K_{\rm s}\int_0^t \omega_{\rm s}\left(l_{\rm s},\tau\right)\mathrm{d}\tau+\pi l_{\rm s}J_{\rm d}\frac{\partial\omega_{\rm s}\left(l_{\rm s},t\right)}{\partial t}=0\,. \tag{31}$$

By using the finite differential method, the angular velocity $\omega_{\rm s}\left(x,t\right)$ of the string is solved from the wave equation (25), initial conditions Eqs. (27) and (28), and boundary conditions Eqs. (30) and (31). With $\omega_{\rm s}\left(x,t\right)$ known, we can determine the helical angle of the threads as

$$\theta_{\rm t}(x,t)=\arctan\left[r_{\rm t0}\frac{\partial}{\partial x}\int_0^t \omega_{\rm s}\left(x,\tau\right)\mathrm{d}\tau\right]. \tag{32}$$

At the end of the winding process, the rotational speed of the disk decreases to 0 .

Further we consider the second case: $M_{\rm local}=0$. The local torque of the string could remain 0 in the case of a sufficiently high release rate ($\left|\dot{\varepsilon}_{\rm s}\right|>\left|\dot{\varepsilon}_{\rm cr}\right|$). Substituting $M_{\rm local}=0$ into Eq. (19) leads to $\partial\omega_{\rm s}/\partial t=0$. From the initial condition Eq. (27), one has $\omega_{\rm s}\left(x\right)=x^m l_{\rm s}^{-m}\omega_{\rm dm}$. Using Eq. (18) and the initial condition $T_{\rm s}\left(x,0\right)=0$, the twist of the string can be solved as

$$T_{\rm s}\left(x,t\right)=\frac{m}{2\pi l_{\rm s}}\left(\frac{x}{l_{\rm s}}\right)^{m-1}\omega_{\rm dm}t\,. \tag{33}$$

With the twist $T_{\rm s}$ known, one can readily determine the geometric configuration (e.g., winding angle and helical angle) of the threads.

Selection of thread materials

Complex deformations of the threads, e.g., tension, bending, and torsion, are involved and coupled in the dynamic process. The internal forces of the threads induced by the bending and torsional deformations are neglected in the above analysis.

This simplification is valid only when the threads are flexible, or in other words, they have relatively low stiffness in both bending and torsion. How to select flexible materials for the threads is revealed by investigating their structure–property relation.

Assume that the deformed threads have a constant helical angle along their length direction. First consider that the thread materials are homogenous and linearly elastic. The strain energy of the two threads due to tensile deformation writes

$$U_{\mathrm{tension}} = E_{\mathrm{t}} A_{\mathrm{t}} \varepsilon_{\mathrm{t}}^2 l_{\mathrm{t}}, \tag{34}$$

where A_{t} and l_{t} denote their cross-sectional area and length, respectively. Using the Euler–Bernoulli beam theory, the bending energy and torsional energy of the threads are written respectively as[8]

$$U_{\mathrm{bend}} = E_{\mathrm{t}} I_{\mathrm{t}} \kappa_{\mathrm{t}}^2 l_{\mathrm{t}}, \tag{35}$$

$$U_{\mathrm{torsion}} = G_{\mathrm{t}} I_{\mathrm{tp}} \tau_{\mathrm{t}}^2 l_{\mathrm{t}} = \frac{E_{\mathrm{t}} I_{\mathrm{tp}} \tau_{\mathrm{t}}^2 l_{\mathrm{t}}}{2\left(1+\nu_{\mathrm{t}}\right)}, \tag{36}$$

where I_{t} and I_{tp} are the moment of inertia and the polar moment of inertia of the cross section, respectively. G_{t} denotes the shear modulus, and κ_{t} and τ_{t} the curvature and torsion of the threads, respectively. For a thread with a circular cross section, we have[9]

$$A_{\mathrm{t}} = \pi r_{\mathrm{t}}^2, \tag{37}$$

$$I_{\mathrm{t}} = \frac{1}{2} I_{\mathrm{tp}} = \frac{1}{4} \pi r_{\mathrm{t}}^4, \tag{38}$$

$$\kappa_{\mathrm{t}} = \frac{\sin^2 \theta_{\mathrm{t}}}{r_{\mathrm{t}}}, \tag{39}$$

$$\tau_{\mathrm{t}} = \frac{\sin \theta_{\mathrm{t}} \cos \theta_{\mathrm{t}}}{r_{\mathrm{t}}}. \tag{40}$$

Substituting Eqs. (37)–(40) into (34)–(36) leads to

$$\frac{U_{\mathrm{bend}}}{U_{\mathrm{tension}}} = \frac{\sin^4 \theta_{\mathrm{t}}}{4\varepsilon_{\mathrm{t}}^2}, \tag{41}$$

$$\frac{U_{\text{torsion}}}{U_{\text{tension}}} = \frac{\sin^2 2\theta_{\text{t}}}{16\varepsilon_{\text{t}}^2 \left(1+\nu_{\text{t}}\right)}. \tag{42}$$

The maximal helical angle θ_{t} of the threads is in the order of 0.1, and the maximal tensile strain ε_{t} is in the order of 0.01. The strain energy U_{bend} and U_{torsion} could be greater than U_{tension}. The internal forces of the threads induced by the bending and torsional deformations will substantially influence the dynamics, which cannot be neglected. For example, during the winding process, the kinetic energy of the disk, in the case of a sufficiently high release rate, is totally converted to the bending and torsional energy of the threads:

$$\frac{1}{4} J_{\text{d}} \omega_{\text{dm}}^2 = U_{\text{bend}} + U_{\text{torsion}}. \tag{43}$$

The maximal winding angle of the threads strongly depends on their bending and torsional stiffness.

Now consider that the thread materials are inhomogeneous. Each thread consists of N_{f} filaments. The moment I_{f} and polar moment I_{fp} of inertia of the filaments are calculated as $I_{\text{f}} = I_{\text{fp}}/2 = \pi r_{\text{f}}^4/4$, where $r_{\text{f}} = r_{\text{t}}/\sqrt{N_{\text{f}}}$ denotes the radius of filament. Assume that all filaments have the same helical angle θ_{t} and helical radius r_{t}. The following relations are then obtained:

$$\frac{U_{\text{bend}}}{U_{\text{tension}}} = \frac{\sin^4 \theta_{\text{t}}}{4N_{\text{f}} \varepsilon_{\text{t}}^2}, \tag{44}$$

$$\frac{U_{\text{torsion}}}{U_{\text{tension}}} = \frac{\sin^2 2\theta_{\text{t}}}{16N_{\text{f}} \varepsilon_{\text{t}}^2 \left(1+\nu_{\text{t}}\right)}. \tag{45}$$

When the filament number N_{f} is sufficiently large, the strain energy U_{bend} and U_{torsion} could be much smaller than U_{tension}. The reduced bending and torsional stiffness of the threads enables an easier winding process. It is therefore preferable to select multi-filament materials (e.g., cotton twine) for the threads. Cotton fibers are $\sim 10^{-5}$ m in diameter[10], and the cotton threads we used in the present study are $\sim 10^{-3}$ m in diameter. The filament number N_{f} could be as large as 10^4, rendering

$U_{\text{bend}}/U_{\text{tension}} \ll 1$ and $U_{\text{torsion}}/U_{\text{tension}} \ll 1$. For the multi-filament threads, it is thus reasonable to neglect the internal forces induced by the bending and torsional deformations.

References

1. Costello, G. A. *Theory of Wire Rope*. (Springer, 1997).
2. Zhao, Z. L., Zhao, H. P., Wang, J. S., Zhang, Z. & Feng, X. Q. Mechanical properties of carbon nanotube ropes with hierarchical helical structures. *J. Mech. Phys. Solids* **71,** 64–83 (2014).
3. Neukirch, S. & Van der Heijden, G. Geometry and mechanics of uniform *n*-plies: from engineering ropes to biological filaments. *J. Elast.* **69,** 41–72 (2002).
4. Grosberg, P., Oxenham, W. & Miao, M. The insertion of 'twist' into yarns by means of air-jets. Part II: twist distribution and twist-insertion rates in air-jet twisting. *J. Text. I.* **78,** 204–219 (1987).
5. Miao, M. & Chen, R. Yarn twisting dynamics. *Text. Res. J.* **63,** 150–158 (1993).
6. Postle, R., Burton, P. & Chaikin, M. The torque in twisted singles yarns. *J. Text. Inst. Trans.* **55,** T448–T461 (1964).
7. Bennett, J. M. & Postle, R. The torque generated in single and multi-ply yarns as a function of changes in yarn tension. *Text. Res. J.* **49,** 499–506 (1979).
8. Ji, X. Y., Zhao, M. Q., Wei, F. & Feng, X. Q. Spontaneous formation of double helical structure due to interfacial adhesion. *Appl. Phys. Lett.* **100,** 263104 (2012).
9. Zhao, Z. L., Li, B. & Feng, X. Q. Handedness-dependent hyperelasticity of biological soft fibers with multilayered helical structures. *Int. J. Non-Linear Mech.* **81,** 19–29 (2016).
10. Rollins, M. L. & Tripp, V. W. Optical and electron microscopic studies of cotton fiber structure. *Text. Res. J.* **24,** 345–357 (1954).

Supplementary figure

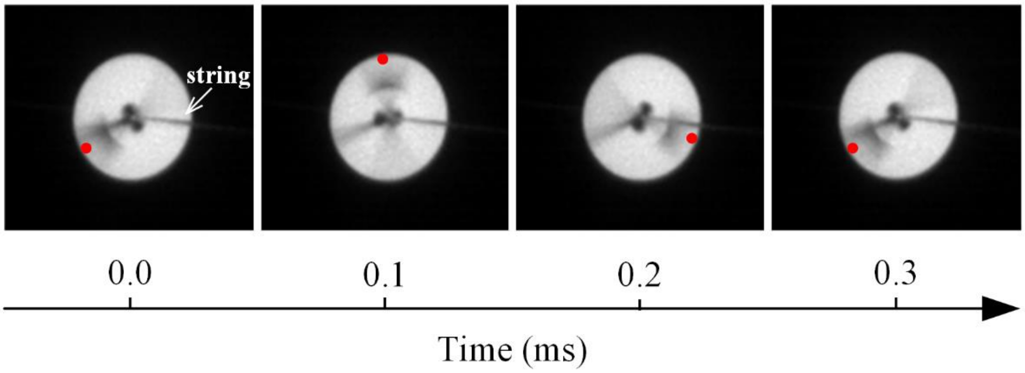

Fig. S1. Time-series graphs of the spinning disk. The rotational speed of the disk was as high as 200,000 rpm.

Supplementary videos

All these videos were taken by the first author Zi-Long Zhao.

S1: **Slow motion analysis of a whirling disk**: Positions of the disk center and the circular marker on the disk were measured. The temporal evolution of the angular velocity of the disk is plotted in Fig. 5a.

S2–S7: **Spinning multiple disks on a string**: There are two disks in S2, three disks in S3 and S4, and four disks in S5–S7. The angular velocities of the disks and their equilibrium positions are illustrated in Fig. 6a–f, respectively.

S8: **Operation of the first electric generator**: The components of the generator are shown in Fig. 7a–c. The video runs at $1/2$ of the actual speed.

S9: **Operation of the second electric generator**: The components of the generator are shown in Fig. 7d–f. The video runs at $1/2$ of the actual speed.
